# Less Is (Often) More: Number of Children and Health Among Older Adults in 24 Countries

**DOI:** 10.1093/geronb/gbad123

**Published:** 2023-08-25

**Authors:** Radoslaw Antczak, Nekehia T Quashie, Christine A Mair, Bruno Arpino

**Affiliations:** Institute of Statistics and Demography, SGH Warsaw School of Economics, Warsaw, Poland; Department of Health Studies, College of Health Sciences, University of Rhode Island, Providence, Rhode Island, USA; Department of Sociology, Anthropology, and Public Health, College of Arts, Humanities, and Social Sciences, University of Maryland, Baltimore, Maryland, USA; Department of Statistical Science and Department of Philosophy, Sociology, Education and Applied Psychology, University of Padua, Padua, Italy

**Keywords:** Cross-national, Fertility, Family, Global aging, Well-being

## Abstract

**Objectives:**

Previous evidence about the impact of parenthood on health for older adults is mixed, perhaps due to variation in number of children and context. Higher numbers of children could lead to support or strain, depending on individual and country contexts. Yet, no studies currently exist that examine associations between the number of children and several health indicators among older adults across multiple global regions.

**Methods:**

We analyze cross-sectional data (1992–2017) of 166,739 adults aged 50+ across 24 countries from the Health and Retirement Study family of surveys to document associations between the number of children, treated as a categorical variable, and 5 health outcomes (self-rated health, activities of daily living limitations, instrumental activities of daily living limitations, chronic conditions, and depression). We perform multivariable analyses by estimating logistic regression models for each country and each outcome.

**Results:**

Multiple comparisons between categories of number of children revealed at least 1 significant difference in each country, and a majority of significant differences indicated those with more children had poorer health. The risk of poorer health for parents of multiple children was observed in 15 countries, but in some countries, fewer children predict poorer health. The greatest number of differences was identified for depression and chronic conditions, and very few for functional limitations.

**Discussion:**

We observe a greater probability that more children are associated with poorer health in later life, especially for chronic conditions and depression. However, a universal global or regional pattern could not be identified. These findings raise new questions about how country contexts shape fertility and health.

Against the backdrop of global demographic shifts—including fertility declines and population aging—a plethora of research has emerged that examines linkages between fertility and later-life health (Grundy & Kravdal, 2010; Grundy et al., 2019; Hurt et al., 2006; Jaffe et al., 2009; Keenan & Grundy, 2019; Sironi et al., 2020). As children are an important source of support for most aging individuals across the globe, a larger family size could increase older adults’ support resources, especially as health declines with age. Yet, having many children can also produce economic, social, emotional, and even biological strains that accumulate over the life course, thereby increasing risks for poor health (Grundy & Read, 2012, 2015). Furthermore, potential health (dis)advantages of having (few or many) children can vary cross-nationally due to heterogeneous informal and formal support to families (Deindl & Brandt, 2017; Hansen et al., 2009), and sociocultural norms about family size (Wu & Penning, 2019).

To date, however, few studies have undertaken a cross-national examination of the associations between the number of children and health, especially across multiple health outcomes and countries (Keenan & Grundy, 2019). Despite some preliminary evidence on this topic, we are unaware of any studies that examine associations between the number of children and health across multiple health outcomes with a cross-national perspective that incorporates multiple countries and regions. To address this gap, we examine cross-national heterogeneity in associations between number of children and the health of adults aged 50 years and older across five health dimensions and 24 middle- and high-income countries in different global regions (i.e., North America, Latin America, Asia, and Europe). We document cross-national variation in the direction and magnitude of older adults’ health as a function of the number of children in different countries.

## Number of Children, Later-Life Health, and Country Context

Number of children may be associated with older adults’ health through several underlying and complex mechanisms, which sometimes appear to result in conflicting findings. First, individuals select into parenthood by various life-course factors that could be confounded with later-life health. For instance, although some studies find that healthier individuals typically have larger families (Hurt et al., 2006; Read et al., 2011), other studies find that early-life socioeconomic disadvantage (e.g., poverty, lower education) is associated with larger family size (Grundy & Read, 2015; Kravdal, 2014). Early-life disadvantages in health and education are associated with different career and family trajectories—especially, for women (Arpino et al., 2018). For parents, childrearing may induce or exacerbate economic challenges, including labor market and economic strains, especially for mothers who experience career disruptions (Gibb et al., 2014). Although the impact of career disruptions varies by country (Anxo et al., 2007), having more children can lead to less time allocated to employment, thus increasing the gender pay gap and potentially accumulating to produce financial strains and health consequences over the life course (Koropeckyj-Cox et al., 2007). From a biological perspective, having (many) children may deplete assets to maintain healthy functioning over the life course due to physical strains of childbirth, stress, lack of sleep, or weight gain (Grundy & Read, 2015; Westendorp & Kirkwood, 1998). Yet, pregnancy and childbirth may be protective against some health conditions, such as breast and reproductive cancers among women (Grundy & Kravdal, 2010).

Challenges associated with larger family sizes may, in some instances, be offset by the psychosocial benefits of childbearing and rearing. Parenthood may trigger individuals to adopt healthier lifestyles, for example, stop smoking (Hank, 2010; Umberson et al., 2010). Older parents may also benefit from support provided by one or more children, and larger family size is associated with an increased chance of regular social contact (Grundy & Read, 2012), which enhances parents’ health (Barefoot et al., 2005; Zunzunegui et al., 2001). Children, however, potentially narrow opportunities for fulfillment in other social roles and present role strain (Evenson & Simon, 2005), which may be a lasting health risk for parents of larger families (Ruppanner et al., 2019).

Finally, the potential later-life health (dis)advantages of having (many) children earlier in the life course are conditioned by the broader socioeconomic contexts in which individuals are embedded. For example, the potential costs and psychosocial strains associated with childrearing vary based on family and community resources, welfare state support (Keenan & Grundy, 2019), and social norms about families (Arpino et al., 2021).

## Empirical Findings on Number of Children and Health

Existing evidence on associations between number of children and later-life health examines a myriad of outcomes, including mortality, physical health (e.g., self-rated health, disability, grip strength), circulatory/metabolic (e.g., chronic conditions), and mental health—with the bulk of studies utilizing samples of older adults in developed countries (Barclay et al., 2016; Grundy & Kravdal, 2010; Grundy & Read, 2015; Hank, 2010; Hinkula et al., 2006; Hurt et al., 2006; Kravdal et al., 2012; Read et al., 2011; Sironi et al., 2020). Studies generally show a U- or J-shaped pattern whereby older adults with low (childless and few children) and high (many children) number of children demonstrate a higher risk of poor health compared to those with two or three children.

There is also evidence that the association between the number of children and health association is not universal, as patterns vary depending on country context and health outcomes. Studies of English older adults suggest that four or more children are associated with poorer health for women and men (Grundy & Read, 2015; Read et al., 2011). In Norway and Finland, however, more children is either unrelated to health or associated with lower mortality (Grundy & Kravdal, 2010; Hinkula et al., 2006), whereas more children are associated with a greater risk of mortality in Sweden (Barclay et al., 2016). In some contexts, a larger family size may be health protective. Hank (2010) found that more children were associated with better self-rated health in West Germany, but poorer physical health for women in East Germany. Yet, Keenan and Grundy (2019) found that European parents with four or more children had worse health across several dimensions (physical, psychological, cognitive) using a cross-national sample of European older adults.

In the United States, women aged 50 and older who had six or more completed pregnancies reported worse health (self-rated and physical functioning) compared to women with fewer than six children, including no children (Kingston et al., 1997). Cultural differences may also shape associations between the number of children and health. White American mothers (aged 50+) with more children were at lower risk for poor mental health (van den Broek, 2021), yet Mexican American women (aged 65+) with six or more pregnancies had more physical mobility limitations than those with four or fewer (Aiken et al., 2012). Finally, African American women with more children had poorer overall health (Sudha et al., 2006), yet other studies found no significant association between the number of children and disability among Black and White mothers (Spence, 2008).

Beyond Europe and the United States, literature on the number of children and later-life health in middle-income countries is burgeoning. Chinese women with more children were more likely to report severe disability, chronic conditions, and worse overall health (Li et al., 2018; Shen et al., 2015). Other studies among Chinese older adults, however, found that more children was associated with better self-rated health among mothers, and better cognitive health for parents (Chen & Lei, 2009). Research from Mexico found that older adults with larger family sizes (e.g., six or more children) have more depressive symptoms and chronic conditions (Diaz-Venegas et al., 2017).

Given that most existing studies suggest that more children tend to be associated with poorer later-life health, having fewer children may confer more health benefits. Yet, empirical evidence about this pattern is also inconclusive. In studies of one or two high-income countries, lifetime childlessness was associated with higher risks of poor health and mortality among older women in England and Wales (Grundy & Tomassini, 2005); higher mortality among Swedes (Barclay et al., 2016), and higher risk of disability among older British women (Read et al., 2011).

In studies of multiple countries using one or two health outcomes, being a parent of one child (for men especially) was associated with depressive symptoms in Eastern Europe (Grundy et al., 2019). Studies of a larger set of European countries using Survey of Health Ageing and Retirement (SHARE) data found mixed results depending on the health outcomes examined. For example, having children (especially two) was associated with lower depression compared to childless (Buber & Engelhardt, 2008; Hank & Wagner, 2013), whereas other studies showed no association with depression (Gibney et al., 2017) or long-term illnesses (Sironi, 2019).

To our knowledge, only one study examined many countries and multiple health outcomes to find evidence of both higher and lower risks of poor health (Quashie et al., 2021). Using data on samples of older adults in Europe, United States, China, and Mexico, the authors found that childlessness was generally unrelated to older adults’ health. Childless older adults had better health in some contexts (e.g., Mexico, Hungary) and worse in others (e.g., Italy). These analyses, however, focused on the presence or absence of children and did not investigate associations between the number of children or compare categories of number of children by country.

Therefore, overall, the existing literature provides conflictual evidence and a blurred picture of the connection between the number of children and health in later-life cross-nationally, including patterns that may vary by country and health dimensions. Nevertheless, there is clear evidence of variation across country contexts, suggesting the need for further cross-national studies with large numbers of countries.

## Current Study Contributions

This study investigates associations between number of children and older adults’ health across five dimensions (self-rated health, disability, activities of daily living [ADL] limitations, instrumental activities of daily living [IADL] limitations, chronic conditions, and depression). We adopt a cross-national perspective to compare links between the number of children (childless to four or more children) and the probability of poor health in 24 high- and middle-income countries. We expect variation in the magnitude and direction of associations between the number of children and health across countries and health outcomes. We treat number of children as a categorical variable. Distinct from previous studies, we compare all possible levels of number of children in terms of health outcomes rather than use a fixed reference category.

## Method

### Data

Our study draws on cross-national harmonized data from the global family of the Health and Retirement Study (HRS) provided by the Gateway to Global Ageing repository (g2aging.org). Our current analysis relies on four studies covering the United States (HRS); Mexico (Mexican Health and Aging Study, MHAS); Europe and Israel (SHARE); and China (China Health and Retirement Study, CHARLS). Altogether, this provides us with 24 high- and middle-income countries. As all these surveys are longitudinal, the datasets include multiple observations (e.g., 13 within the HRS) for most respondents. Our pooled dataset uses the first-observation approach wherein we select the first observation of each respondent, after pooling all waves in each dataset. This approach allows us to focus on relationships between number of children, which is an almost perfectly time-invariant measure in our sample of older adults, and health at older ages, while maximizing available sample sizes. Although the surveys vary in sampling strategies, interview methods, and respondent selection, the participation rates for the baseline and refreshment samples were generally high (Bergmann et al., 2022; Wong et al., 2017) thereby ensuring representativeness.

As the HRS studies vary in the minimum age of respondents (e.g., 45 for CHARLS, 50 for all others), we restrict our sample to respondents aged 50 years and older for comparability. Further, we exclude 11,720 (6.6%) observations with missing values on any of the explanatory or outcome variables. This provides a final analytic sample of 166,739 individuals from 1992 to 2017. Supplementary Materials present country samples by waves.

### Measures

Following previous research (Quashie et al., 2021), we adopt a multidimensional approach to health that includes five indicators of physical and mental health: self-rated health, ADL limitations, IADL limitations, chronic conditions, and depression. Our selection of health measures was driven by the need of using both general and specific measures, comparability across surveys, and previous research. We dichotomize all outcome variables with 1 = poor health, 0 = good health. Our main explanatory variable, number of children, is a categorical variable representing the number of living children at the time of interview including biological/step/adopted children (0 = ref, 1, 2, 3, 4, and more). This is the most harmonized measure of family size across all the datasets used in our current study. Although the variable does not directly measure fertility history (number of children ever born) nor distinguish between biological and nonbiological children, we are still able to assess the importance of older adults’ family size to later-life health conditions. Furthermore, by assessing all living children we capture the role of social control and support that children may exert on older adults’ health.

Our cutoff of four or more children partly reflects the distribution of our data (11.4% of our total sample had more than four children, with only four countries reporting a higher share of parents with more than four children, with a maximum of 47% in Mexico) and is consistent with prior research (Grundy et al., 2019). We also include covariates for individual characteristics known to be associated with parenthood and health, including gender, age (continuous), partnership status, education level, total assets (quartiles), and location of residence (urban/rural). Table 1 provides an overview of all variables included in our analysis.

**Table 1.** Description of Variables Used in the Multivariate Analyses

Variable	Description (survey question)	Measurement	Categories (reference category in bold)
Self-Rated Health	Respondents’ self-reported health, scale excellent to poor	Dichotomous	**Good**, Poor
Activities of Daily Living (ADL)	Wallace Scale, 0–3. Derived variable counting number of at least some difficulties in bathing, dressing or eating	Dichotomous	**No ADL limitations**, At least 1
Instrumental Activities of Daily Living (IADL)	Constructed using difficulty with managing money, taking medications, shopping, preparing meals	Dichotomous	**No IADL limitations,** At least 1
Chronic conditions	Constructed using ever diagnosed with high blood pressure, diabetes, cancer, stroke, lung disease, and heart disease	Dichotomous	**No conditions,** At least 1 condition
Depression	Single-item measure: Felt depressed in the week prior to the interview	Dichotomous	**Not depressed,** Depressed
Number of children	Number of living children	Continuous	0, 1, 2, 3, 4, and more
Age	Respondents’ age	Continuous	Years
Gender	Respondents’ sex	Dichotomous	**Men**, women
Marital status	Current marital status	Categorical	**Partnered**, widowed, separated/divorced/never married
Location of residence	Respondent’s living region	Categorical	**Urban**, Rural
Education	Harmonized education levels	Categorical	**Less than lower secondary,** upper Secondary and vocational, tertiary
Total household assets quartile	Total household assets, adjusted for household size	Categorical	**Q1,** Q2, Q3, Q4, No answer

*Note*: In the case of Mexico, respondents’ urban–rural residence was determined by merging the MHAS Wave 3 data with the 2015 Master file to determine the household’s location of residence. Following the methods of Salinas et al. (2010), urban areas combine locations with population size 15,000–99,999 (semi-urban) and 100,000 and more (urban). Rural households combine locations with population size of 2,500–14,999 (semi-rural) and those <2,500 (rural).

### Analytic Strategy

First, we present descriptive statistics of our key variables by country (Table 2). Second, we perform multivariable analyses by estimating separate logistic regression models for each country to assess associations between number of children and each health outcome. For every country and health outcome, we compare each of the five categories of number of children to the other categories of number of children. Thus, each country (24 total) has 20 comparisons of the categories of number of children, which is repeated across five health outcomes and yields 2,400 comparisons (20 × 24 × 5).

**Table 2.** Distribution of Explanatory (number of children) and Outcome (five health measures) Variables

		Austria	Belgium	China	Croatia	Czechia	Denmark	Estonia	France	Germany	Greece	Hungary	Ireland
Number of children	Childless	12.6	12.2	2.6	8.2	5.6	8.2	8.5	10.5	12.6	11.0	8.9	16.2
	1 child	21.6	20.3	15.4	20.3	17.7	13.6	23.9	18.0	23.1	17.9	22.7	6.6
	2 children	35.0	34.3	31.0	51.7	51.0	42.8	41.8	35.2	37.6	49.8	47.3	21.4
	3 children	17.6	19.3	23.7	14.5	18.4	22.2	16.4	20.7	17.5	16.2	15.2	18.8
	4 and more children	13.2	13.9	27.3	5.4	7.3	13.2	9.43	15.3	9.0	4.9	5.8	36.8
Self-rated health	Good	70.5	72.6	22.2	56.1	56.0	78.0	30.6	65.3	61.7	72.4	39.0	78.2
	Poor	29.5	27.3	77.7	43.8	43.9	21.9	69.3	34.6	38.2	27.5	60.9	21.8
ADL	No ADL	91.9	87.7	88.7	92.1	91.8	93.1	86.3	89.6	91.7	94.0	88.8	89.8
	At least 1 ADL	8.1	12.2	11.3	7.8	8.2	6.9	13.6	10.3	8.2	6.0	11.1	10.1
IADL	No IADL	92.2	90.6	78.4	93.2	92.6	93.3	87.9	92.1	93.2	93.5	84.1	91.0
	At least 1 IADL	7.8	9.3	21.5	6.8	7.3	6.6	12.0	7.9	6.7	6.5	15.8	9.0
Chronic conditions	No conditions	44.4	40.3	36.8	36.1	32.6	41.6	29.8	40.7	39.2	45.9	24.6	48.8
	At least 1 chronic condition	55.5	59.6	63.1	63.8	67.4	58.3	70.2	59.2	60.7	54.0	75.3	51.1
Depression	No symptoms	65.9	60.0	46.8	58.3	60.8	69.1	48.0	52.6	57.4	63.2	59.3	69.9
	Any depressive symptoms	34.0	39.9	53.1	41.6	39.1	30.8	51.9	47.3	42.5	36.7	40.7	30.0
Sample	*N*	5,962	9,235	13,387	2,393	8,074	5,438	7,278	7,605	8,346	5,903	2,946	991
		Israel	Italy	Luxembourg	Mexico	The Netherlands	Poland	Portugal	Slovenia	Spain	Sweden	Switzerland	USA
Number of children	Childless	4.0	13.2	11.4	5.3	10.2	6.2	7.6	6.3	10.6	7.1	14.4	11.0
	1 child	11.7	22.2	20.0	5.4	10.7	12.4	22.7	19.7	16.8	13.9	14.2	13.7
	2 children	25.1	40.6	41.6	11.9	42.3	38.0	42.3	53.1	37.2	40.7	39.2	27.4
	3 children	27.6	15.8	17.8	15.8	22.0	23.5	15.5	15.3	19.3	23.0	20.2	19.3
	4 and more children	31.4	8.1	9.1	61.5	14.6	19.7	11.8	5.5	15.8	15.2	11.7	28.4
Self-rated health	Good	60.8	61.6	67.4	39.0	72.9	41.8	37.8	58.4	59.7	81.5	82.8	69.4
	Poor	39.1	38.3	32.5	60.9	27.0	58.1	62.1	41.5	40.3	18.5	17.1	30.5
ADL	No ADL	88.4	91.2	89.7	90.8	94.4	83.2	84.7	91.4	90.2	93.2	94.8	86.8
	At least 1 ADL	11.5	8.7	10.3	9.1	5.6	16.7	15.2	8.5	9.8	6.8	5.2	13.1
IADL	No IADL	84.6	92.3	92.7	93.8	93.7	85.7	88.1	92.6	90.6	94.1	96.3	86.4
	At least 1 IADL	15.3	7.7	7.3	6.2	6.3	14.2	11.8	7.3	9.4	5.9	3.7	13.5
Chronic conditions	No conditions	40.6	41.9	32.6	45.0	49.1	33.7	32.7	39.0	40.1	44.2	51.0	30.8
	At least 1 chronic condition	59.3	58.0	67.3	54.9	50.8	66.3	67.2	60.9	59.8	55.7	48.9	69.1
Depression	No symptoms	59.3	62.0	54.2	64.4	67.6	42.9	50.5	61.9	63.4	68.0	59.2	83.3
	Any depressive symptoms	40.6	37.9	45.7	35.5	32.3	57.0	49.4	38.0	36.5	32.0	40.7	16.6
Sample	*N*	3,543	7,984	2,009	16,120	6,177	2,938	2,038	5,078	8,248	6,404	4,346	24,296

*Notes*: ADL = activities of daily living; IADL = instrumental activities of daily living.

**Figure 1.**
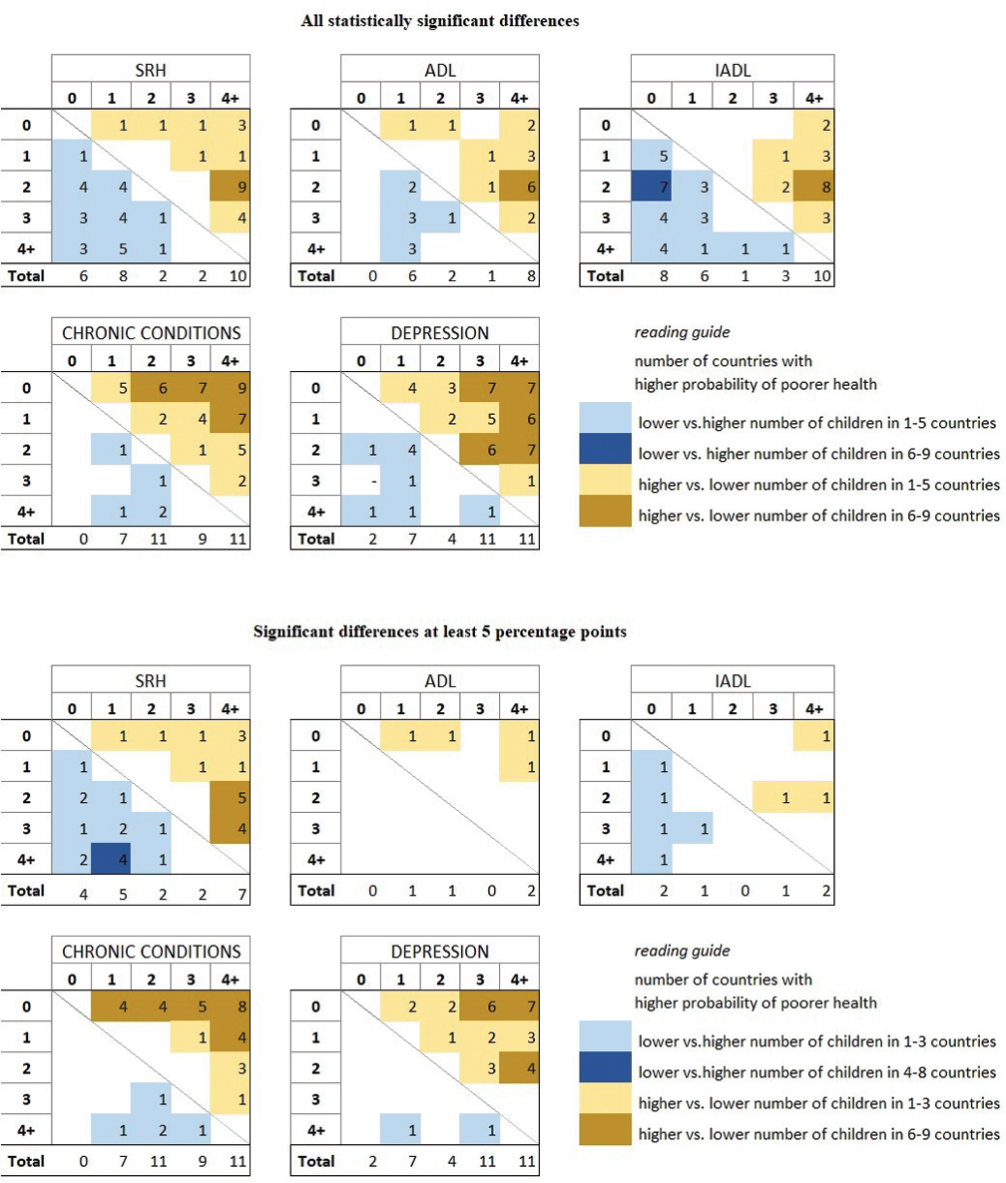
Summary of statistically significant differences between categories of number of children and health outcomes showing the total number of countries.

**Figure 2.**
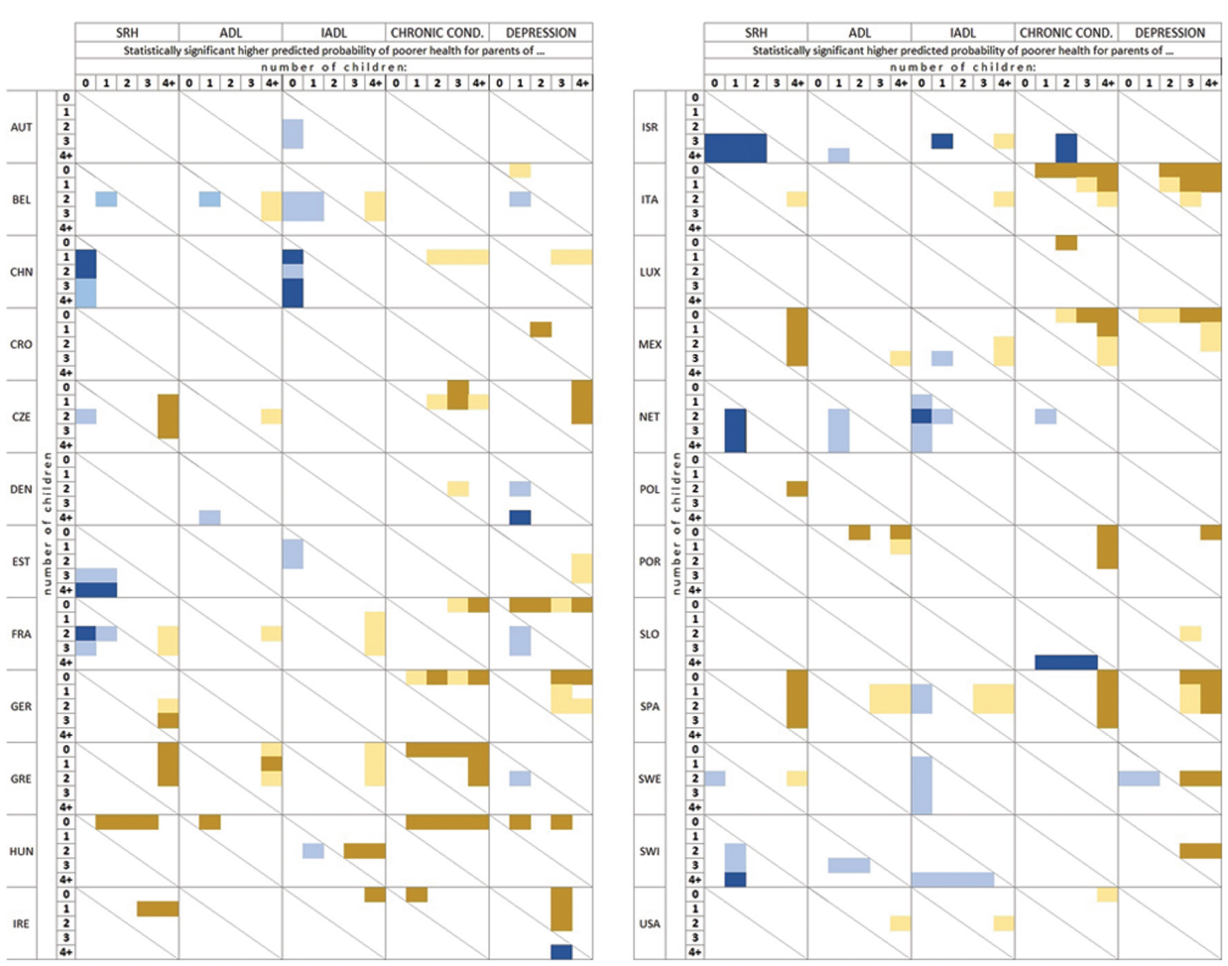
Graphical illustration of statistically significant differences in predicted probabilities of poorer health between categories of number of children. Reading guide for Figure 2. The figure presents the comparison in probability of poor health between all pairs of number of children. Shaded squares identify statistically significant difference between a pair of number of children: one in a column, one in a row, that is, the category in a column is associated with poorer health compared to a category in a row. Country codes AUT (Austria), BEL (Belgium), CHN (China), CRO (Croatia), CZE (Czechia), DEN (Denmark), EST (Estonia), FRA (France), GER (Germany), GRE (Greece), HUN (Hungary), IRE (Ireland), ISR (Israel), ITA (Italy), LUX (Luxembourg), MEX (Mexico), NET (Netherlands), POL (Poland), POR (Portugal), SLO (Slovenia), SPA (Spain), SWE (Sweden), SWI (Switzerland) and USA (United States of America).

To identify health outcomes more clearly by number of children, our multivariable analysis included comparisons between each level of the number of children variable in terms of their resulting predicted probabilities. The extensive fully adjusted results with predicted probabilities are presented in Supplementary Tables. The main text includes a reader-friendly visual presentation of the results. Specifically, we conducted statistical tests of differences among pairs of estimates (predicted probabilities) by estimating 84% confidence intervals so that the corresponding significance level for pairwise comparisons is about 5% (Macgregor-Fors & Payton, 2013). Figure 1 presents these results by counting the number of countries with statistically significant differences, and Figure 2 displays the results by country. This visual approach allows us to display which category of number of children is significantly different from the others. All statistically significant differences in Figure 2 are shaded and differences greater than 5 percentage points (pp) are in a darker color to highlight differences that are substantial.

Existing studies show that fertility has different impacts on women and men, which also differs by age; therefore, we additionally run separate analyses by gender and age groups (50–64 and 65+).

## Results

### Descriptive

Table 2 presents distributions of our outcome variables and number of children, separately by country. Although most countries show a median of two children (except higher in Ireland, Israel, and Mexico), the distribution of number of children varies cross-nationally. Health outcomes also vary by country. The share of older adults who report poor self-rated health ranges from 17.1% in Switzerland to 77.8% in China. ADL and IADL limitations have less variation, with the prevalence of having 1 + ADL limitations ranging from 5.2% in Switzerland to 16.8% in Poland, and 1 + IADL limitations ranging from 3.7% in Switzerland to 21.5% in China. Yet, most older adults in the sample report at least one chronic condition, ranging from 49% in Switzerland to over 75% in Hungary. The final health indicator, depression, ranges from 16.7% in the United States, to 30%–45.7% in Western Europe, and a maximum of 57.1% in Poland.

### Multivariable Results

Our multivariable analysis aimed to identify which category of number of children poses a statistically significant higher probability of poorer health within each country. Although we analyze all possible comparisons between single categories of number of children, to ease the interpretation, we focus on identifying whether higher versus lower number of children, or lower versus higher, poses a greater risk of poorer health. Higher versus lower number of children means 4+ children versus 3, 2, 1, and 0; 3 children versus 2, 1 and 0; 2 children versus 1, and 0 children; and 1 child versus childless. Lower versus higher number of children means the inverse comparisons.

Of our total 2,400 comparisons, only 237 (10%) were statistically significant at the 5% level, including 117 (5%) differences that were at least 5 pp, which we consider to be more substantial. Among all statistically significant differences, 158 (67%) were related to a greater risk of poorer health for higher versus lower number of children, and the remaining 79 (33%) for lower versus higher number of children. Among the substantial (5+ pp) and statistically significant differences, however, 90 (77%) were identified for higher versus lower number of children and only 27 (23%) for lower versus higher number of children. Overall, the greatest number of statistically significant differences among health outcomes were noted for depression (58, including 32 substantial), and the lowest for ADL (26, including 4 substantial). We also observe great heterogeneity among countries in associations between the number of children and our five health outcomes: from only one statistically significant difference in Luxembourg, Croatia, and Poland to 24 significant differences in Spain.

In the following sections, we present detailed results. First, we present the number of countries (out of 24) with statistically significant differences in associations between the number of children and health. Second, we present significant differences by health measure and number of children. Our presentation of results focuses on substantial and statistically significant differences (at least 5 pp). These differences are presented in dark colors: dark yellow for higher versus lower number of children, and dark blue for lower versus higher number of children.

#### Number of countries with significant differences

Figure 1 presents the number of countries with statistically significant differences in the predicted probabilities at the 5% level between the categories of number of children, separately for each health measure. In total, we observed substantial (at least 5 pp) and statistically significant differences in 21 countries. In three countries (Austria, Belgium, United States) all differences were below 5 pp.

Across all health measures, 15 countries (63% of all analyzed countries) showed a greater probability of poorer health for higher number of children versus lower number of children (dark yellow color), and 9 countries (38%) for lower number of children versus higher number of children (dark blue color). Three countries—France, Ireland, Switzerland—had evidence of poor health for both higher and lower number of children.

Considering differences by number of children and health outcome, we observed that greater risk of poor health for higher versus lower number of children (yellow color) occurred more often: for depression in 12 countries, chronic conditions in 11 countries, and self-rated health in eight countries. For ADL and IADL, substantial differences were identified only in single countries. The highest number of countries where lower number of children predicted greater risk of poorer health (blue color) was noted for self-rated health (six countries), and in single countries for other health measures, excluding ADL.

#### Results by health measure and number of children

Figure 2 presents statistically significant differences between categories of number of children and poor health using predicted probabilities. Shaded squares indicate statistically significant probabilities, with darker colors indicating substantial differences in predicted probabilities (at least 5 pp). For example, in Belgium, light orange in column “4+” indicates that parents of four or more children (in a column) have a higher probability of having at least one ADL limitation versus parents of two and three children (in a row). In China, the dark blue in column “0” indicates that childless older adults have a higher probability of poorer SRH versus parents of one and two children, and these differences are substantial (at least 5 pp). The diagonal line indicates noncomparable differences (i.e., 1 vs 1 or 3 vs 3 children), and white spaces present nonstatistically significant differences.

##### SRH.

Higher versus lower number of children predicts poorer self-rated health in eight countries: Czechia, Germany, Greece, Hungary, Ireland, Mexico, Poland, and Spain. These differences (all but Hungary) are related to four or more children, suggesting that parents of four or more in these countries are at higher risk of poor SRH compared to parents of less than four (usually one or two). In two countries, this disadvantage also applies to parents of less than four children: parents of three, two, and one child versus childless in Hungary, and parents of three and two children versus one child in Ireland.

In six countries, we observed poorer self-rated health for lower versus higher number of children. No children versus other categories predicted poorer SRH in four countries (China, Estonia, France, and Israel). Having one child versus other categories also predicts poorer SRH in four countries (Estonia, Israel, The Netherlands, and Switzerland). In Israel only, parents of two versus three, and four or more children showed higher risks of poor SRH.

##### ADL.

Higher versus lower number of children (four or more children vs childless or one child) predicted having at least one ADL in three countries (Greece, Hungary, and Portugal). In Hungary, having one child was associated with having ADL limitations compared to being childless. We did not observe any substantial statistically significant differences for lower versus higher number of children.

##### IADL.

Higher versus lower number of children was associated with a higher risk of having IADL limitations in two countries (Hungary and Ireland). In both cases, parents of four or more children had a higher probability of IADL limitations versus childless (Ireland) and parents of two children (Hungary). In Hungary, parents of three versus two children had a greater risk of IADL limitations.

Lower versus higher number of children was associated with IADL limitations in three countries (China, Israel, and The Netherlands). No children versus other categories were related to greater IADL risk in two countries (China, childless vs one, three, and four or more children; The Netherlands, childless vs two children). In Israel, parents of one child had a higher risk of IADL limitations.

##### Chronic conditions.

Higher versus lower number of children predicted greater risk of having at least one chronic condition in 11 countries (Czechia, France, Germany, Greece, Hungary, Ireland, Italy, Luxembourg, Mexico, Portugal, and Spain). These patterns include risk among those with four or more children (vs childless) in eight countries and those with one child (vs childless) in five countries. We also observed the pattern of poorer health of all parents versus childless in three countries (Greece, Hungary, and Italy).

Risk of at least one chronic condition for lower versus higher number of children was observed only in two countries (higher risk among fewer than three children in Israel and among childless in Slovenia).

##### Depression.

Greater risk of depression for higher versus lower number of children was found in 12 countries, the highest number of countries compared to other health measures, including Croatia, Czechia, France, Germany, Hungary, Ireland, Italy, Mexico, Portugal, Spain, Sweden, and Switzerland. Higher risks of depression were evident mainly among parents of three (eight countries) or four or more children (nine countries) compared to childless (and less often, parents of one child).

We found a higher probability of depression for lower versus higher number of children in only two countries—Denmark (one child vs four or more) and Ireland (three vs four or more).

##### Additional analyses: models by gender and age groups.

Supplementary Figures 3–6 present the results of models by gender and age groups. As expected, a number of children have different associations with health in subgroups. However, when assessing the overall picture, that is, number of countries with significant associations, the variation by subgroups is visible in specific cases. The most striking differences by gender groups identified are women with multiple children have more chronic conditions than mothers of few children or childless women in more countries than fathers; but in the case of depression, fathers of multiple children are more disadvantaged in more countries than mothers. Additionally, women with few children (usually one) have poorer self-rated health than men with few children.

Age group differences showed that older parents (65 years and older) of multiple children have a higher probability of chronic conditions than parents from the younger age group (50–64). However, younger parents (50–64) of multiple children have poorer self-rated health and higher risk of depression than older parents. Additionally, younger parents of one child or childless have a higher risk of IADL than those in the older age group.

## Discussion

The number of children shapes an individual’s health in myriad ways across the life course. Yet, the potential health (dis)advantages associated with the number of children vary according to the type of health measure considered as well as country contextual factors. Few existing studies examine the association between the number of children and multiple dimensions of health cross-nationally, and where available, the studies focus on cross-national variation within one global region: Europe (Gibney et al., 2017; Keenan & Grundy, 2019; Sironi, 2019).

Using cross-national harmonized data representing older adults from diverse global regions (North America, Latin America, Europe, and Asia), our study adopts a nuanced approach with comparisons across five categories of the number of children with attention to comparing pairs of the levels of the number of children across a sample of 24 countries and five health dimensions.

The results of our analysis yield complex patterns across numbers of children, health outcomes, and countries. Specifically, we find that (1) in most countries, higher numbers of children are associated with poorer health; (2) of the five health outcomes examined, depression was most commonly associated with higher numbers of children, and (3) substantial cross-national variation exists to the extent that regional patterns could not be identified. Taken together, these findings suggest that higher numbers of children do not appear to be beneficial for health in a majority of countries—especially in terms of mental health—and it is difficult to identify any regional or income-based pattern to the cross-country differences observed, underscoring the need for significantly more cross-national studies on this topic.

### Higher Number of Children and Worse Health in Most Countries

Overall, among the statistically significant and substantial differences, a higher number of children predicted poorer health in most (77%) of cases. Consistent with prior studies (e.g., Diaz-Venegas et al., 2017; Grundy & Read, 2015; Li et al., 2018; Sudha et al., 2006), we found that having more children was associated with poorer health among older adults across diverse contexts. Although many previous studies have used a fixed reference category of the number of children, such as childless or having two children, we offer a methodological advancement by comparing pairs of the categories of the number of children. Some prior research using a fixed reference category of two children, suggests that having two children is protective for parents’ physical and mental health relative to being childless or having more than two children. However, our findings suggest that detailed comparisons of the categories of number of children, provide no clear “happy medium” for parents’ later-life health. Rather, a greater number of children regardless of the specific number (e.g., 3 vs 0, 2 vs 1, etc.) is more often associated with poor health. Although less common, we also found that having fewer children is associated with higher risks of poor health in some contexts.

Thus, the association between the number of children and health is complex. Although having more children can mean more potential social control or support resources in later life, adult children are not guaranteed to be available to offer support due to multiple factors including geographic proximity or role strain of managing multiple social roles and responsibilities. Furthermore, having more and fewer (though less often for the latter) children is associated with higher probabilities of poor health and may reflect various social selection factors that precede childbearing. These factors may include the poorer health and lower socioeconomic resources of parents prior to childbearing, or a potentially smaller social network of parents with fewer children.

Complexity is even greater when adding subgroups of older people to our examinations. Mothers of multiple children are more disadvantaged by chronic conditions than men, whereas fathers of multiple children are at greater risk of depression than mothers. Younger parents (50–64 years old) of multiple children have a greater risk of poor self-rated health and depression than older parents. Our results also reveal substantial variation across health outcomes and country contexts, described in the next sections.

### Mixed Findings by Health Outcomes, Clearest Links to Depression

Higher number of children was most clearly linked to poorer health when examining depression, but with substantial variation across countries. Previous studies found conflicting findings, with either higher or lower number of children predicting the risk of depression or no statistically significant association. Similar to findings from Mexico (Diaz-Venegas et al., 2017) and Europe (Kruk & Reinhold, 2014), only for women (Keenan & Grundy, 2019), we found that a higher number of children were associated with depression in these contexts. Higher depression risk for parents of multiple children might be an indirect effect of fertility histories, such as early parenthood, short birth intervals, or difficult financial situations that occur more frequently with a high number of children (Grundy et al., 2020).

Consistent with previous cross-national studies of European older adults (Grundy et al., 2019; Hank & Wagner, 2013), we also found evidence of lower number of children and depression in certain European contexts—namely Denmark and Ireland—and virtually no statistically significant relationships in the United States (Sudha et al., 2006). It is challenging to compare these conflicting patterns across contexts, as there appears to be no discernible pattern by economic development or welfare regime. These associations may reflect more idiosyncratic mediating psychosocial factors such as strained relations with children (Grundy & Read, 2015) and social selection into parenthood whereby those with fewer children have a poorer lifetime (mental) health that shapes the low number of children–depression linkage (Buber & Engelhardt, 2008).

Likewise, for chronic conditions, higher number of children predicts poorer health more often than lower number of children, with parents of four or more children appearing to be particularly disadvantaged. This pattern aligns with the results of previous studies in various countries (e.g., Europe: Keenan & Grundy, 2019; China: Shen et al., 2015; Mexico: Diaz-Venegas et al., 2017), potentially reflecting the combined influence of physiological depletion from multiple childbearing and rearing as well as role strains that challenge maintaining a healthy lifestyle. Additionally, gender differences are also important when analyzing chronic conditions. A cross-national study using a sample of European older adults found significant associations between high number of children and long-term illness only among women in Austria and France (Sironi, 2019), which was also confirmed in our study.

Yet, we also found that having multiple children was associated with a lower risk of chronic conditions in Slovenia and Israel, which is not observed elsewhere in the literature. It is possible that having multiple children in these contexts is health protective due to strong social norms of intergenerational support and wider community ties, which may also extend from having larger families (Hrast et al., 2020; Tur-Sinai et al., 2020). These countries should be examined further in future work due to this unique pattern and the challenges to sustained reliance on family ties as population age.

In the case of self-rated health, similar numbers of countries showed significant differences for higher versus lower number of children, and lower versus higher number of children. These mixed findings are also consistent with previous literature. For example, there is moderate evidence that high number of children is a disadvantage in the United States (Sudha et al., 2006) and China (Li et el., 2018), but higher number of children is an advantage in contexts such as West Germany (Hank, 2010).

Finally, number of children was weakly associated with the probability of functional limitations (IADL and ADL) in most countries in our study. Existing evidence showed more limitations of parents of multiple children (e.g., Li et al., 2018; Read, Grundy, & Wolf, 2011), but our results did not confirm this relationship, even for the same contexts (such as China).

### Substantial and Nonpatterned Variation Across Country Contexts

In addition to variation across health outcomes, we observe substantial variation between countries. Yet, we cannot point to any specific broad contextual links between fertility and later-life health—similar associations can be found in high- or mid-income countries, in Western and Eastern Europe, and across welfare regimes and cultural (e.g., religion) contexts. However, we identify special country cases worth further investigation.

The first interesting case is the United States, where the link between fertility and older adults’ health is very weak (no substantial differences). Hence, here any potential health benefits/disadvantages of higher or lower number of children are likely moderated by other factors, such as sociocultural differences, for example, ethnicity, as identified in previous studies (Aiken et al., 2012; Spence, 2008; van den Broek, 2021). For example, van den Broek (2021) found lower risk of mental health for White mothers of multiple children, whereas Spence (2008) found that having multiple children was not significantly associated with the physical and mental health of White and Black mothers. Thus, the mixed evidence in the fertility–later-life health relationship among U.S. adults may also reflect methodological differences across study designs and samples.

Second, in Israel, all substantial associations were related to the disadvantages of lower number of children (having one or two children is associated with health risk compared to three or more). This finding somewhat confirms previous studies showing higher mortality risks of childless women and mothers of one child, perhaps linked to nonpregnancy biological factors as well as psychosocial and lifestyle factors (Jaffe et al., 2009). Similarly, in China, childless women have poorer health than parents (but only for two health measures), suggesting the negative implications of aging without traditional social support resources in societies with strong cultural values on children alongside limited formal infrastructure to support older adults (Wu & Penning, 2019).

In addition, Hungary is the only country with substantial health risk of higher number of children across all health measures. In Hungary, having multiple children may be a widespread health disadvantage.

Finally, there is a group of countries with clear health risks of higher number of children (four or more children) for at least three measures, mainly from Southern Europe: Greece, Portugal, and Spain (and Italy for two measures), but also including Mexico and Czechia. These patterns suggest possible contextual social and economic conditions including (limited) social welfare family benefits that might cause strains, particularly for those with large families.

### Limitations and Future Research

Our analyses show a complex pattern of associations between number of children and health cross-nationally. Although we used nationally representative samples, some of the sub-samples (for categories of number of children) are relatively low (such as parents with 1 child in Ireland, *n* = 65 or parents with 4+ children, *n* = 130). Therefore, some of the insignificant associations might the result of low sample size. Additionally, missing data on variables leading to the exclusion of 6.6% of the observations might be not at random, thereby undermining the representativeness of some country samples. Relatedly, systemic disparities in the underreporting of civil registration within China (Goodkind, 2011; Yang et al., 2022) and Mexico (Harbers, 2020) potentially contribute to overestimation bias for these countries. Underreporting of chronic conditions due to lack of knowledge or diagnoses may also be an issue in these data. Additionally, due to the cross-sectional nature of our analyses, we cannot disentangle the direction of associations between number of children and health. Furthermore, our models include control variables that may also mediate or be confounded with fertility histories and lifestyle choices (e.g., education, household income). Unfortunately, however, our harmonized data do not provide information on early-life experiences such as socioeconomic conditions during the childbearing years, health behaviors, and limitations during the childrearing years, all of which may help elucidate family experiences—such as selection into parenting, resource strain, and health outcomes. Finally, we were unable to investigate the role of macro-level confounders in this analysis, which could contribute to understanding the relationship between fertility and health, especially, in this cross-country comparison. Future research should prioritize thorough explorations of macro-level predictors in the association between number of children and health.

## Conclusion

Higher number of children is far from being a universal risk, however, having higher numbers of children is more often connected with health risks, especially for chronic conditions and depression. Yet, this association was present in only half of the analyzed countries. The picture for other health dimensions is blurred, and in most countries, number of children is not associated with health.

The patterns identified in our analyses are not necessarily causal but raise several questions for future research to understand the underlying risk factors faced by aging adults cross-nationally. We encourage future studies to examine the role of individual and contextual mechanisms (e.g., supportive versus strained relations with children, the relative importance of children’s gender—number of sons versus daughters, early life-course health behaviors, and availability of social welfare supports) that may elucidate the conditions that shape existing linkages between family size and later-life health in diverse populations across the globe.

## Supplementary Material

gbad123_suppl_Supplementary_Material
