# Supplementary material for: Less Is (Often) More: Number of Children and Health Among Older Adults in 24 Countries
Source: J Gerontol B Psychol Sci Soc Sci. 2023 Aug 25;78(11):1892–902. doi: 10.1093/geronb/gbad123 (PMC10645313; doi:10.1093/geronb/gbad123)
Supplement: gbad123_suppl_Supplementary_Material [file gbad123_suppl_supplementary_material.pdf]

Table 3. Number of observation by country and survey wave

wave number	country, survey												
	Austria	Belgium	China	Croatia	Czechia	Denmark	Estonia	France	Germany	Greece	Hungary	Ireland	Israel
	SHARE	SHARE	CHARLS	SHARE	SHARE	SHARE	SHARE	SHARE	SHARE	SHARE	SHARE	SHARE	SHARE
1	1505	3642	11883	0	0	1583	0	2826	2881	2577	0	0	2240
2	65	307	1232	0	2595	1286	0	861	978	830	0	991	0
3	0	0	0	0	0	0	0	0	0	0	0	0	719
4	4232	2830	272	0	3974	440	6541	3407	50	0	2946	0	0
5	136	1406	0	0	1409	1868	177	183	4375	0	0	0	552
6	24	1049	0	2393	95	261	560	328	62	2491	0	0	32
7	0	1	0	0	1	0	0	0	0	5	0	0	0
8	0	0	0	0	0	0	0	0	0	0	0	0	0
9	0	0	0	0	0	0	0	0	0	0	0	0	0
10	0	0	0	0	0	0	0	0	0	0	0	0	0
11	0	0	0	0	0	0	0	0	0	0	0	0	0
12	0	0	0	0	0	0	0	0	0	0	0	0	0
13	0	0	0	0	0	0	0	0	0	0	0	0	0
	5962	9235	13387	2393	8074	5438	7278	7605	8346	5903	2946	991	3543

wave number	country, survey											
	Italy	Luxembourg	Mexico	Netherlands	Poland	Portugal	Slovenia	Spain	Sweden	Switzerland	USA	Total
	SHARE	SHARE	MHAS	SHARE	SHARE	SHARE	SHARE	SHARE	SHARE	SHARE	HRS	
1	2467	0	11183	2780	0	0	0	2222	2945	937	0	51671
2	1154	0	125	833	2378	0	0	865	655	725	7223	23103
3	0	0	4403	0	0	0	0	0	0	0	133	5255
4	1393	0	409	842	205	1910	2670	1804	102	2558	4217	40802
5	1768	1570	0	1722	0	0	951	3194	2599	89	142	22141
6	1202	439	0	0	354	128	1457	161	103	37	152	11328
7	0	0	0	0	1	0	0	2	0	0	2649	2659
8	0	0	0	0	0	0	0	0	0	0	129	129
9	0	0	0	0	0	0	0	0	0	0	101	101
10	0	0	0	0	0	0	0	0	0	0	5314	5314
11	0	0	0	0	0	0	0	0	0	0	233	233
12	0	0	0	0	0	0	0	0	0	0	126	126
13	0	0	0	0	0	0	0	0	0	0	3877	3877
	7984	2009	16120	6177	2938	2038	5078	8248	6404	4346	24296	166739

Table 4. Distribution of Explanatory (number of children) and Outcome (5 health measures) Variables. Women.

		Austria	Belgium	China	Croatia	Czechia	Denmark	Estonia	France	Germany	Greece	Hungary	Ireland
Number of children	childless	12.2	11.6	1.9	7.8	5.0	7.3	8.8	9.7	11.5	11.3	8.2	14.9
	1 child	23.4	21.3	14.6	21.0	18.4	14.6	25.8	19.6	23.5	18.9	24.1	6.2
	2 children	34.3	34.1	30.0	51.1	50.9	42.4	41.5	34.4	37.8	48.1	46.6	20.3
	3 children	16.8	18.9	23.8	14.3	18.6	22.3	15.5	20.6	17.8	16.5	15.6	19.2
	4 and more children	13.4	14.2	29.7	5.8	7.1	13.5	8.4	15.8	9.4	5.3	5.6	39.5
Self-rated health	good	70.5	71.2	19.3	53.9	55.2	76.7	30.2	64.5	61.6	67.7	35.9	76.7
	poor	29.5	28.8	80.7	46.1	44.8	23.3	69.8	35.5	38.4	32.3	64.1	23.3
ADL	no ADL	91.6	86.3	87.0	91.1	91.2	92.9	86.1	89.7	91.3	92.9	89.0	89.4
	at least 1 ADL	8.4	13.7	13.0	8.9	8.8	7.1	13.9	10.3	8.7	7.1	11.0	10.6
IADL	no IADL	91.6	88.8	74.3	90.9	92.1	92.4	87.7	90.9	92.5	91.7	80.6	89.9
	at least 1 IADL	8.4	11.2	25.7	9.1	7.9	7.7	12.3	9.1	7.5	8.3	19.4	10.1
Chronic conditions	no conditions	44.3	39.3	33.5	34.1	32.1	40.1	27.7	39.1	40.3	43.1	22.1	47.5
	at least 1 chronic condition	55.8	60.7	66.5	65.9	67.9	59.9	72.4	60.9	59.7	56.9	77.9	52.5
Depression	no symptoms	59.7	52.0	41.5	49.5	53.8	62.6	41.4	43.1	49.9	54.6	52.3	65.4
	any depressive symptoms	40.3	48.0	58.5	50.5	46.2	37.4	58.6	56.9	50.1	45.4	47.7	34.6
Sample	N	3 417	4 921	6 780	1 320	4 602	2 863	4 311	4 195	4 374	3 172	1 658	537

		Israel	Italy	Luxembourg	Mexico	Netherlands	Poland	Portugal	Slovenia	Spain	Sweden	Switzerland	USA
Number of children	childless	4.1	13.4	10.7	5.1	10.0	4.6	8.4	5.6	10.1	6.7	14.1	10.9
	1 child	12.8	22.2	20.7	6.1	10.7	13.0	22.6	19.9	17.2	15.1	14.9	14.7
	2 children	25.5	39.7	42.4	11.6	41.7	38.6	41.1	52.7	36.9	40.0	39.1	27.5
	3 children	27.8	16.1	17.2	15.2	22.4	23.9	15.8	15.8	19.6	23.3	20.1	19.4
	4 and more children	29.8	8.6	9.0	62.1	15.1	20.0	12.1	6.1	16.2	14.9	11.8	27.6
Self-rated health	good	60.5	58.1	65.1	33.2	72.4	41.0	34.5	57.4	55.3	79.7	81.7	68.2
	poor	39.5	41.9	35.0	66.8	27.6	59.0	65.5	42.6	44.7	20.3	18.3	31.8
ADL	no ADL	87.8	89.8	88.1	89.2	93.5	82.6	82.0	92.0	88.7	93.0	94.8	85.3
	at least 1 ADL	12.2	10.2	11.9	10.8	6.5	17.4	18.1	8.0	11.3	7.0	5.2	14.7
IADL	no IADL	83.6	90.8	91.9	91.8	93.0	83.8	85.2	91.7	89.1	93.4	95.8	84.7
	at least 1 IADL	16.4	9.2	8.1	8.2	7.0	16.2	14.8	8.3	10.9	6.6	4.2	15.3
Chronic conditions	no conditions	42.4	40.9	32.2	37.4	47.8	30.2	31.7	40.0	37.0	44.3	52.8	29.3
	at least 1 chronic condition	57.6	59.1	67.9	62.6	52.2	69.9	68.3	60.0	63.1	55.7	47.2	70.7
Depression	no symptoms	52.3	53.8	46.0	56.0	61.5	35.3	39.3	56.3	53.6	60.5	50.4	80.8
	any depressive symptoms	47.7	46.2	54.1	44.0	38.5	64.7	60.7	43.7	46.4	39.5	49.6	19.2
Sample	N	1 900	4,272	1 073	9 033	3 289	1 602	1 119	2 818	4 411	3 370	2 308	13 748

Table 5. Distribution of Explanatory (number of children) and Outcome (5 health measures) Variables. Men.

		Austria	Belgium	China	Croatia	Czechia	Denmark	Estonia	France	Germany	Greece	Hungary	Ireland
Number of children	childless	13.2	12.9	3.4	8.6	6.3	9.3	8.1	11.6	13.9	10.7	9.8	17.8
	1 child	19.3	19.3	16.2	19.5	16.7	12.5	21.1	16.2	22.8	17.0	21.0	7.1
	2 children	35.9	34.5	31.9	52.4	51.2	43.2	42.2	36.5	37.4	51.9	48.2	22.9
	3 children	18.8	19.8	23.6	14.6	18.2	22.0	17.8	20.9	17.3	15.9	14.9	18.5
	4 and more children	12.9	13.5	24.8	4.9	7.7	13.0	10.9	14.9	8.7	4.5	6.1	33.7
Self-rated health	good	70.5	74.3	25.2	58.9	57.3	79.5	31.3	66.3	61.9	77.9	43.2	80.0
	poor	29.5	25.8	74.8	41.1	42.7	20.5	68.7	33.7	38.1	22.1	56.8	20.0
ADL	no ADL	92.4	89.5	90.4	93.6	92.7	93.4	86.7	89.5	92.3	95.3	88.7	90.3
	at least 1 ADL	7.6	10.5	9.6	6.4	7.3	6.6	13.3	10.5	7.7	4.7	11.3	9.7
IADL	no IADL	93.1	92.8	82.8	96.1	93.4	94.5	88.4	93.7	94.2	95.7	88.7	92.3
	at least 1 IADL	6.9	7.2	17.2	3.9	6.5	5.5	11.6	6.3	5.8	4.3	11.3	7.7
Chronic conditions	no conditions	44.7	41.5	40.3	38.7	33.2	43.3	32.9	42.9	38.1	49.4		50.4
	at least 1 chronic condition	55.3	58.6	59.7	61.3	66.8	56.7	67.1	57.1	61.9	50.6	72.0	49.6
Depression	no symptoms	74.2	69.3	52.4	69.3	70.2	76.5	57.8	64.3	65.7	73.2	68.3	75.3
	any depressive symptoms	25.8	30.7	47.6	30.7	29.8	23.5	42.2	35.7	34.3	26.8	31.7	24.7
Sample	N	2 545	4 314	6 607	1 073	3 472	2 575	2 967	3 410	3 972	2 731	1 288	454

		Israel	Italy	Luxembourg	Mexico	Netherlands	Poland	Portugal	Slovenia	Spain	Sweden	Switzerland	USA
Number of children	childless	3.8	13.2	12.2	5.6	10.5	8.2	6.5	7.1	11.3	7.5	14.8	11.3
	1 child	10.6	22.2	19.2	4.5	10.8	11.8	23.0	19.6	16.5	12.6	13.6	12.4
	2 children	24.8	41.7	40.8	12.5	43.1	37.4	43.7	53.8	37.6	41.5	39.4	27.5
	3 children	27.6	15.5	18.6	16.6	21.5	23.2	15.2	14.7	19.1	22.7	20.6	19.3
	4 and more children	33.3	7.5	9.2	60.9	14.1	19.5	11.5	4.9	15.4	15.6	11.7	29.5
Self-rated health	good	61.2	65.7	70.2	46.6	73.7	42.8	41.9	59.7	64.7	83.5	84.2	71.1
	poor	38.8	34.3	29.8	53.4	26.4	57.2	58.1	40.3	35.3	16.6	15.9	28.9
ADL	no ADL	89.2	93.0	91.6	93.0	95.4	84.1	88.3	90.8	92.0	93.4	95.0	89.0
	at least 1 ADL	10.8	7.0	8.4	7.0	4.6	15.9	11.8	9.2	8.0	6.6	5.1	11.0
IADL	no IADL	85.8	94.1	93.7	96.4	94.6	88.1	91.8	94.0	92.4	94.9	96.9	88.9
	at least 1 IADL	14.2	5.9	6.3	3.6	5.4	11.9	8.2	6.0	7.6	5.1	3.1	11.2
Chronic conditions	no conditions	38.6	43.2	33.2	54.7	50.7	38.0	34.1	37.9	43.9	44.1	49.0	32.9
	at least 1 chronic condition	61.4	56.8	66.8	45.3	49.3	62.1	65.9	62.1	56.1	55.9	51.0	67.1
Depression	no symptoms	67.6	71.6	63.7	75.3	74.6	52.0	64.2	68.9	74.8	76.3	69.3	86.6
	any depressive symptoms	32.4	28.4	36.3	24.7	25.5	48.0	35.8	31.1	25.3	23.7	30.7	13.4
Sample	N	1 643	3 712	936	7 087	2 888	1 336	919	2 260	3 837	3 034	2 038	10 548

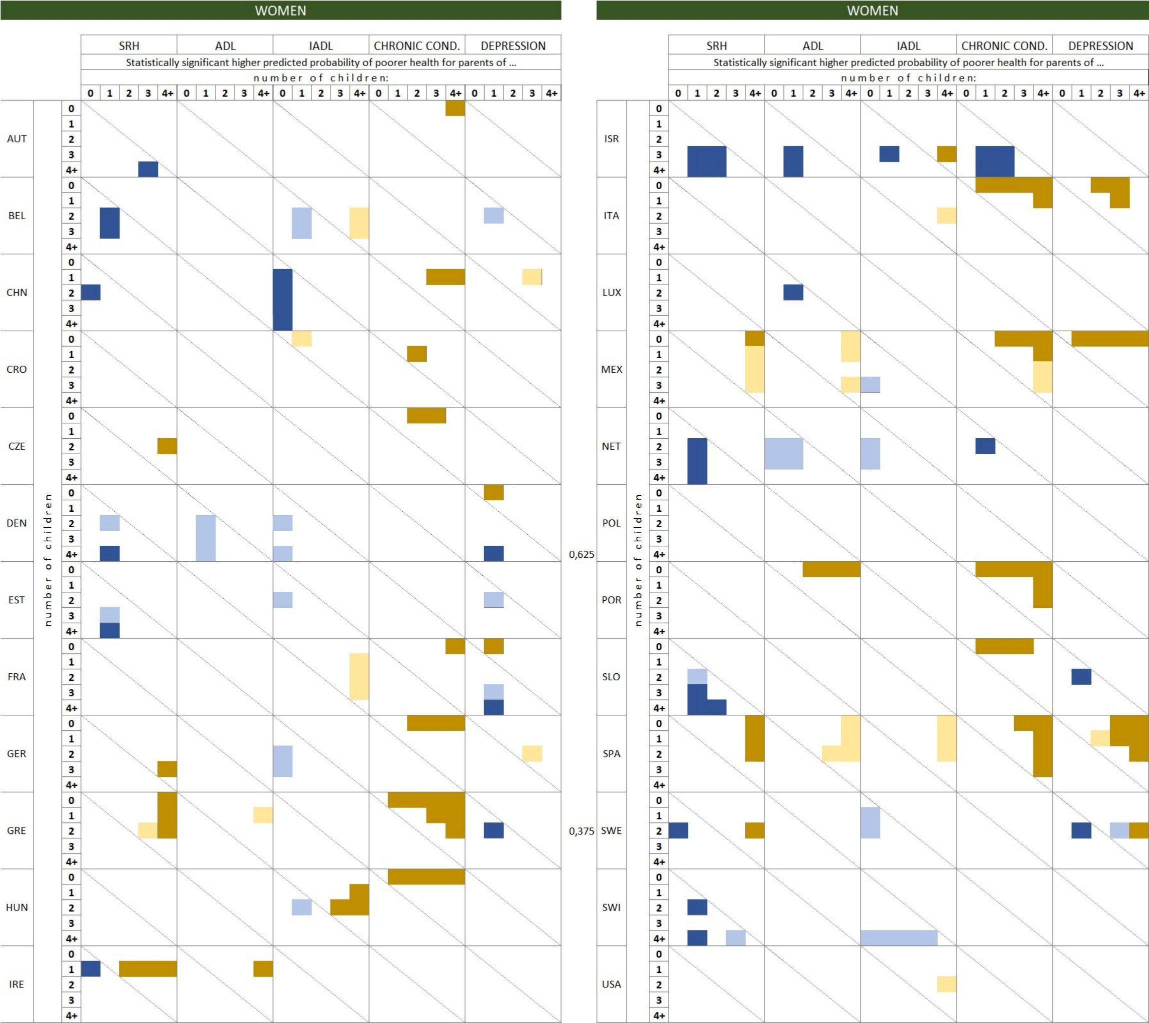
WOMEN
SRH
ADL
IADL
CHRONIC COND.
DEPRESSION
Statistically significant higher predicted probability of poorer health for parents of ...
number of children:
0 1 2 3 4+
number of children
AUT
BEL
CHN
CRO
CZE
DEN
EST
FRA
GER
GRE
HUN
IRE
0,625
0,375
WOMEN
SRH
ADL
IADL
CHRONIC COND.
DEPRESSION
Statistically significant higher predicted probability of poorer health for parents of ...
number of children:
number of children
ISR
ITA
LUX
MEX
NET
POL
POR
SLO
SPA
SWE
SWI
USA

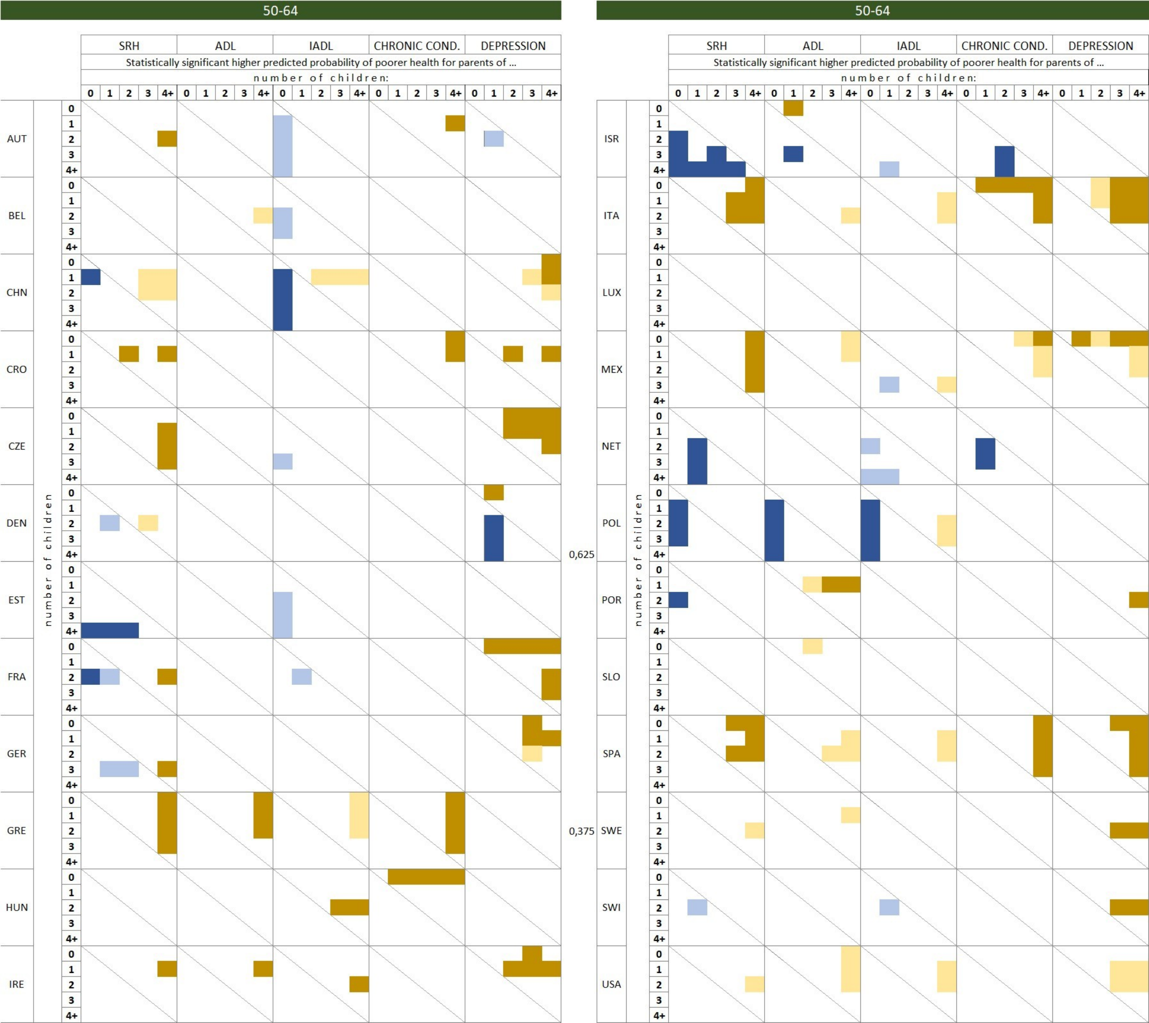

50-64
SRH
ADL
IADL
CHRONIC COND.
DEPRESSION
Statistically significant higher predicted probability of poorer health for parents of ...
number of children:
0 1 2 3 4+
number of children
AUT
BEL
CHN
CRO
CZE
DEN
EST
FRA
GER
GRE
HUN
IRE
0,625
0,375
50-64
ISR
ITA
LUX
MEX
NET
POL
POR
SLO
SPA
SWE
SWI
USA

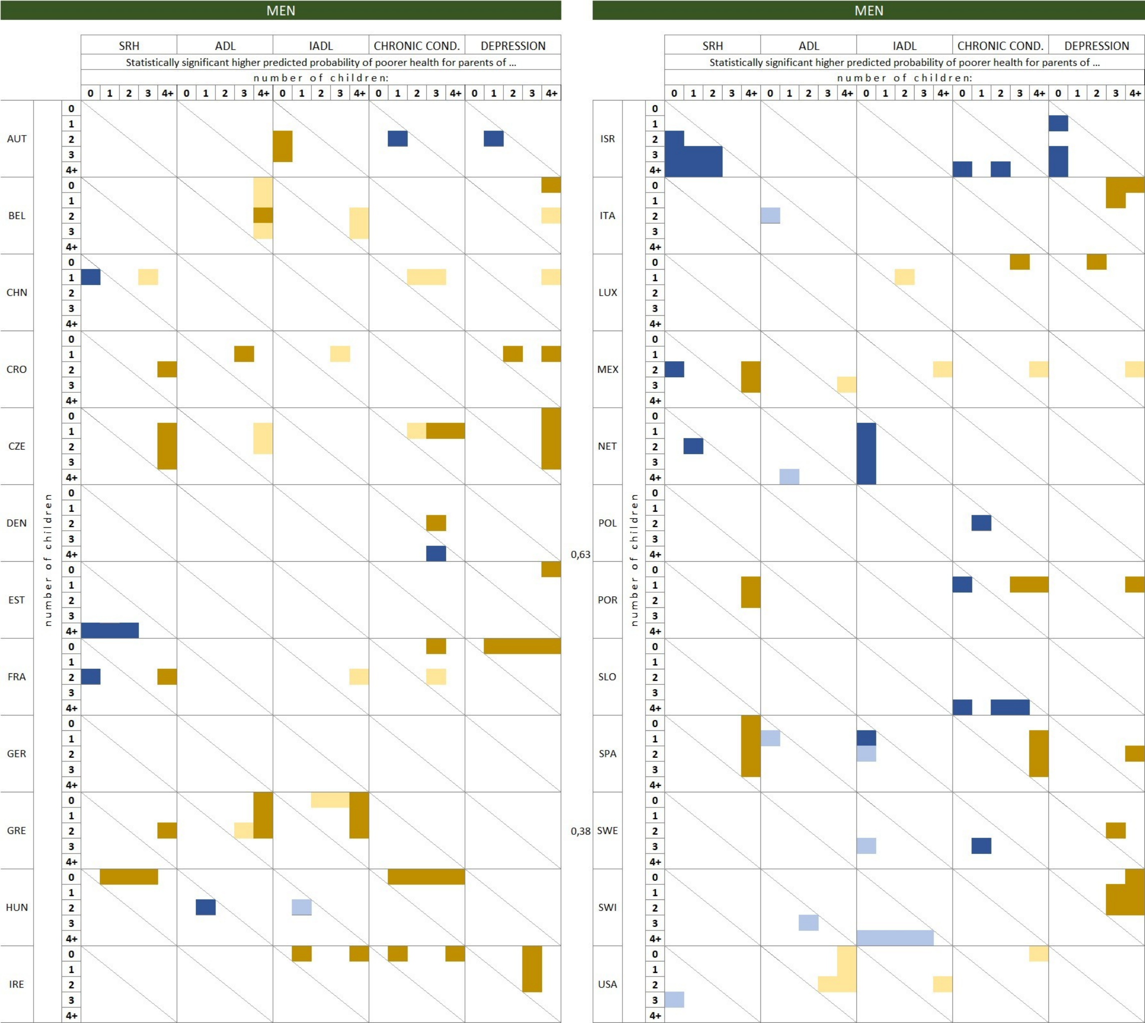
MEN
SRH
ADL
IADL
CHRONIC COND.
DEPRESSION
Statistically significant higher predicted probability of poorer health for parents of ...
number of children:
0 1 2 3 4+
number of children
AUT
BEL
CHN
CRO
CZE
DEN
EST
FRA
GER
GRE
HUN
IRE
MEN
ISR
ITA
LUX
MEX
NET
POL
POR
SLO
SPA
SWE
SWI
USA
0,63
0,38

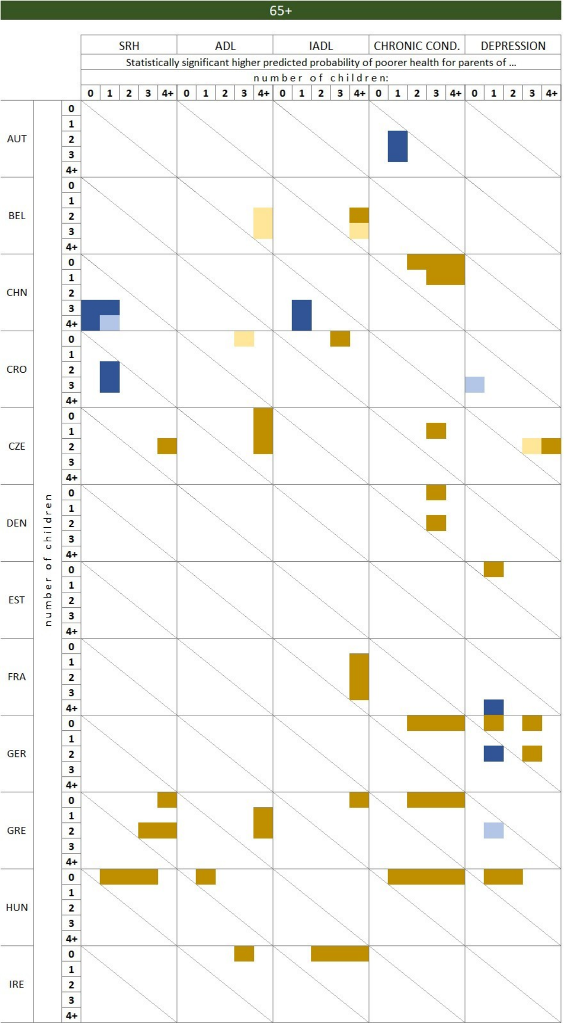
65+
SRH
ADL
IADL
CHRONIC COND.
DEPRESSION
Statistically significant higher predicted probability of poorer health for parents of ...
number of children:
0 1 2 3 4+
number of children
AUT
BEL
CHN
CRO
CZE
DEN
EST
FRA
GER
GRE
HUN
IRE

0,63

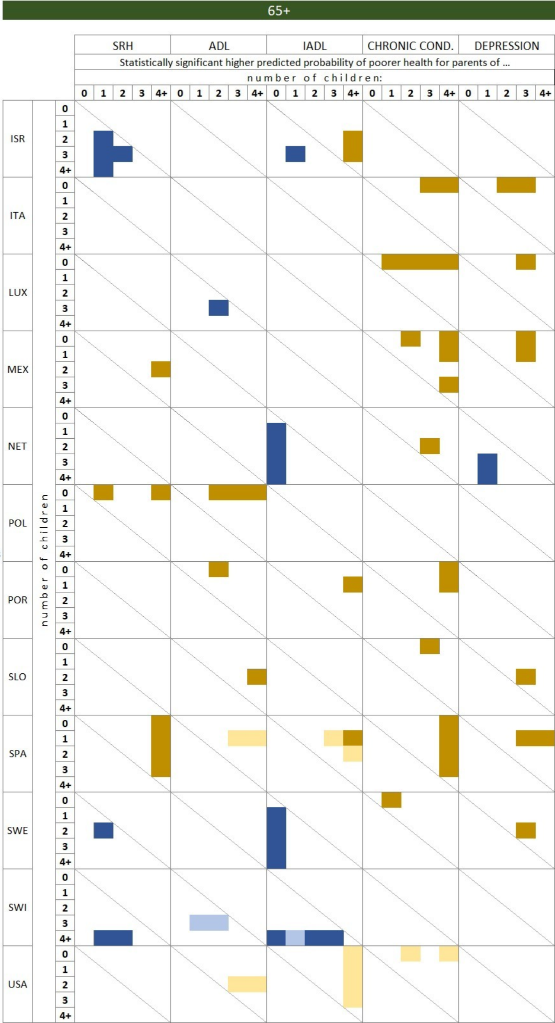
65+
SRH
ADL
IADL
CHRONIC COND.
DEPRESSION
Statistically significant higher predicted probability of poorer health for parents of ...
number of children:
0 1 2 3 4+
number of children
ISR
ITA
LUX
MEX
NET
POL
POR
SLO
SPA
SWE
SWI
USA
